# Supplementary material for: Antiviral Mechanism of Virucidal Sialic Acid Modified Cyclodextrin
Source: Pharmaceutics. 2023 Feb 9;15(2):582. doi: 10.3390/pharmaceutics15020582 (PMC9965221; doi:10.3390/pharmaceutics15020582)
Supplement: Supplementary file 1 [file pharmaceutics-15-00582-s001.zip › pharmaceutics-2094632-SI.docx]

# **Supplementary Materials**

**Antiviral mechanism of virucidal sialic acid modified cyclodextrin**

**Yong Zhu ^1^, Andrey Sysoev ^2^, Paulo Jacob Silva ^1^, Marine Batista ^1^ and Francesco Stellacci ^1^.**

^1^ Institute of Materials, École Polytechnique Fédérale de Lausanne, Switzerland. Station 12, Lausanne 1015, Switzerland

^2^ Department of Mechanistic Cell Biology, Max-Planck Institute of Molecular Physiology, Otto-Hahn-Straße 11, 44227 Dortmund, Germany

* Correspondence: fancesco.stellacci@epfl.ch


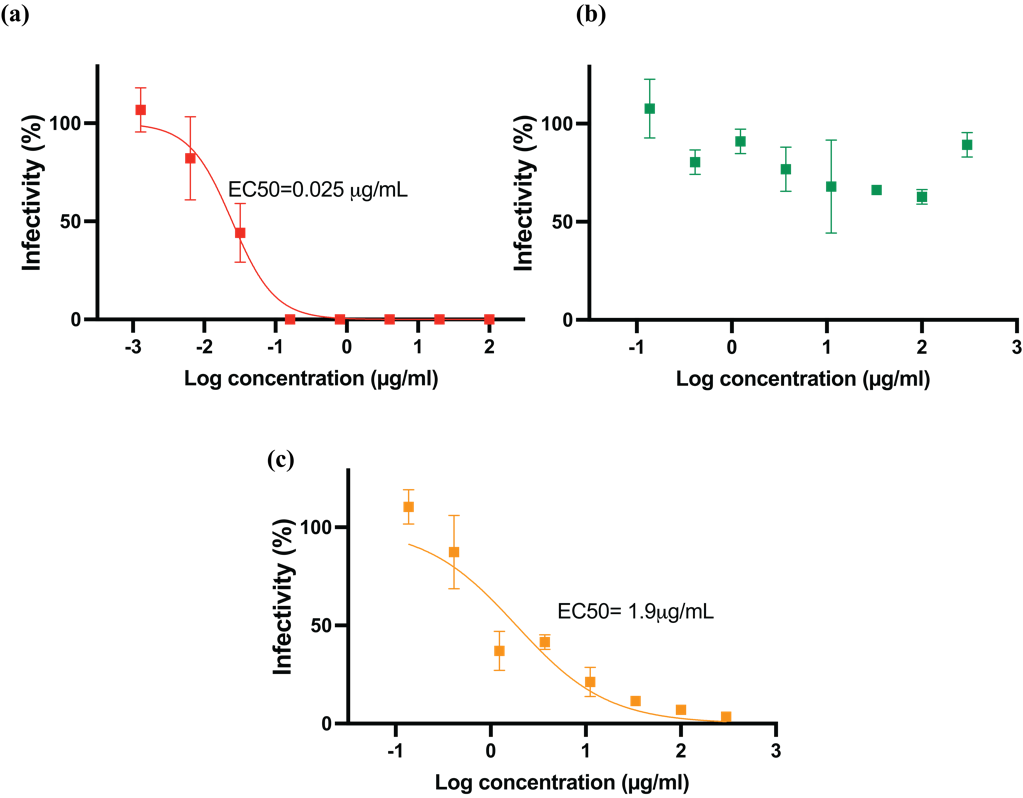


Figure S1. H1N1 Inhibition of (a) CD-6’SLN EC_50_ = 0.025 μg/mL (95% CI 0.017-0.035 μg/mL), (b) CD-(S-C11-COOH)_7_ EC_50_ > 300 μg/mL and (c) CD-(Mal-PEG8)_7_-6’SLN EC_50_ = 1.86 μg/mL (95% CI 1.05-3.42 μg/mL) against H1N1 (A/Netherlands/2009). The virus was first treated with test materials for 1 h and then applied to the cell.

Figure S2. Virucidal results of CD-6’SLN, CD-(S-C11-COOH)_7_ and CD-(Mal-PEG8)_7_-6’SLN against H1N1 (A/Netherlands/2009).


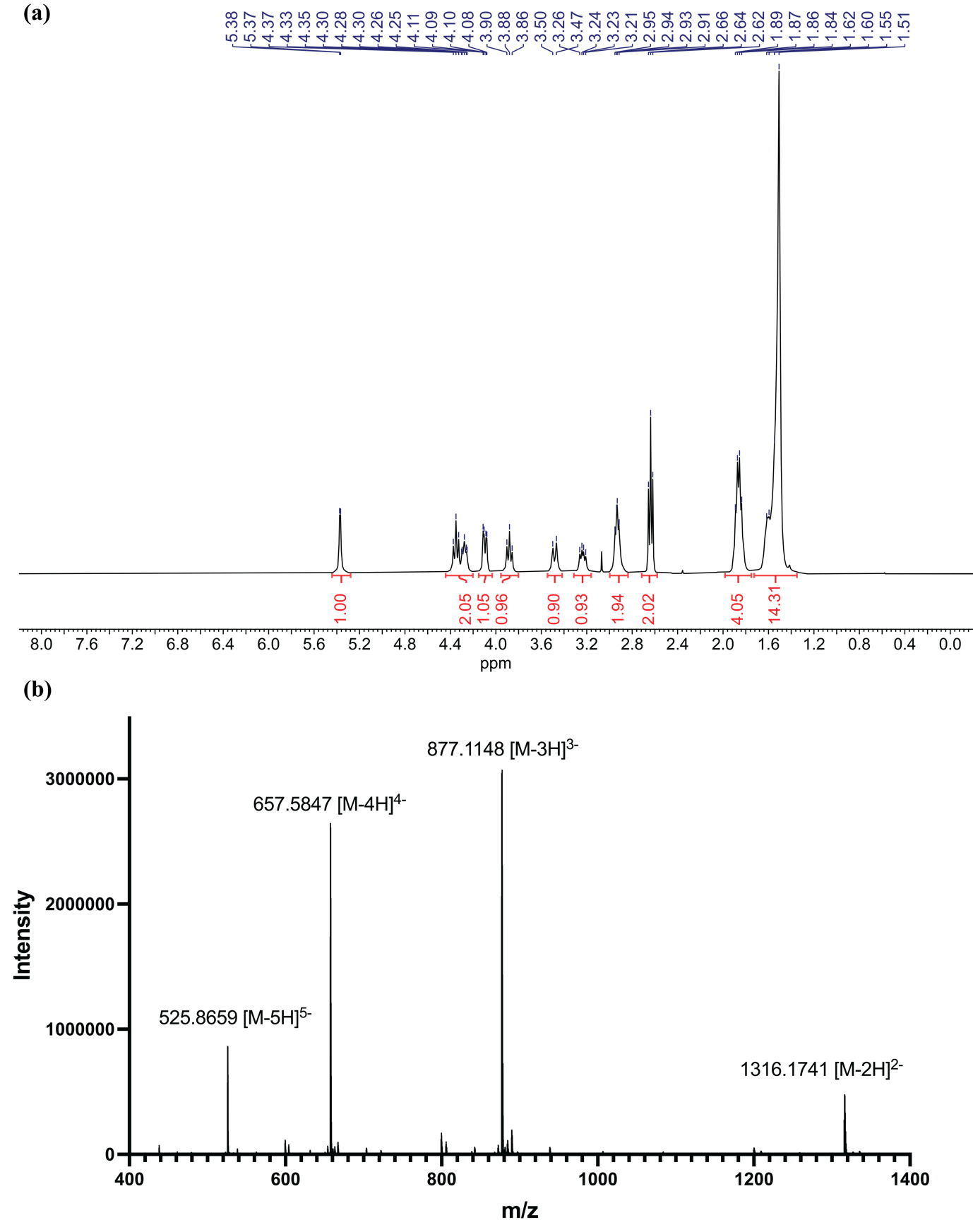
Figure S3. Characterization of CD-(S-C11-COOH)_7_. (a) ^1^H NMR of CD-(S-C11-COOH)_7_ in TFA-d (b) Mass spectrum of CD-(S-C11-COOH)_7_.

Figure S4. ^1^H NMR of CD-6’SLN in D_2_O. Average number of 6’SLN (2.5) per β-cyclodextrin was calculated by comparing the integral of a distinctive peak from trisaccharide (blue arrow) and the integral of a peak from β-cyclodextrin (red arrow). Both peaks represent 1 hydrogen.

Figure S5. DOSY NMR of CD-6’SLN in D_2_O shows the product is free of 6’SLN.


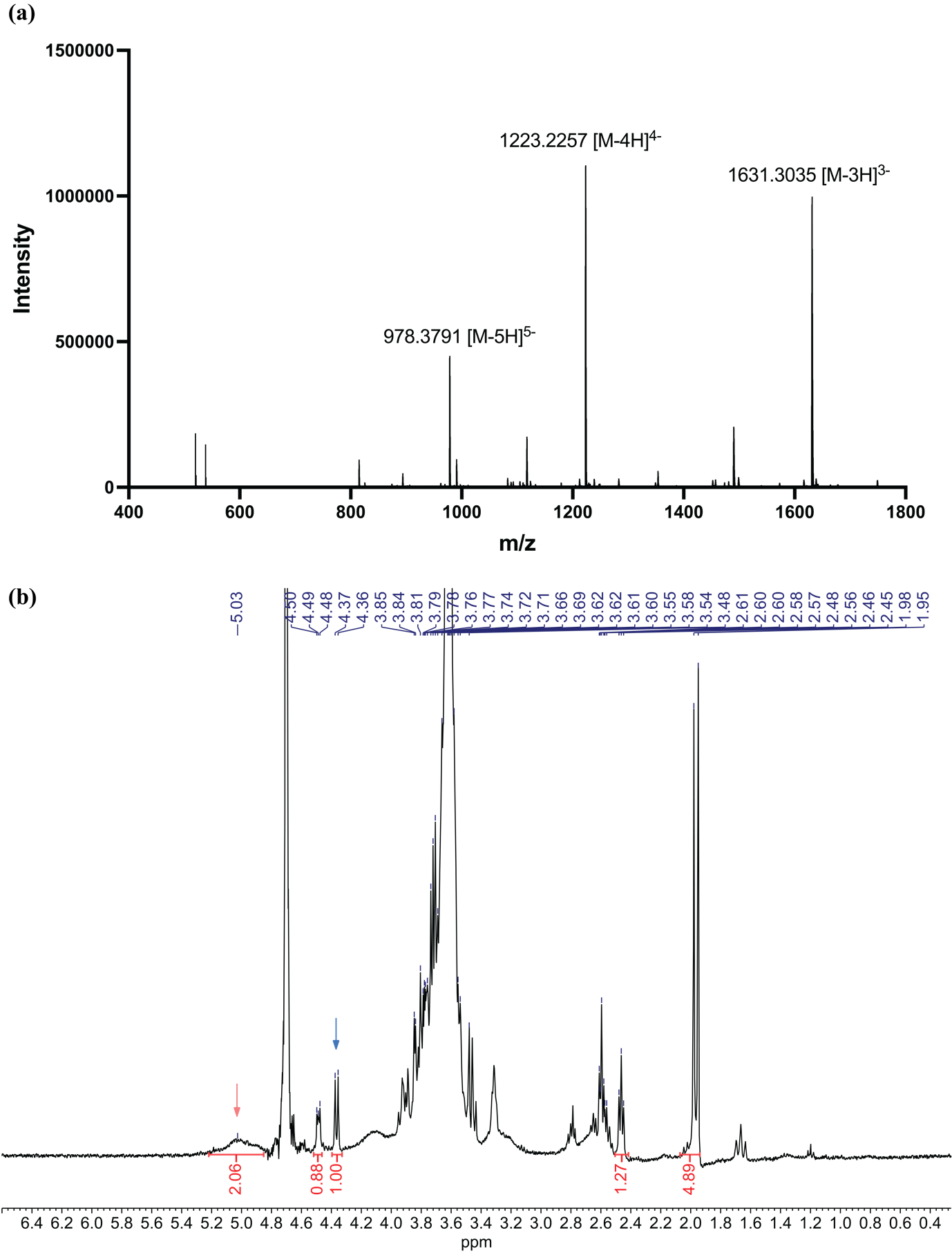


Figure S6. Characterization of CD-(Mal-PEG8)_7_-COOH and CD-(Mal-PEG8)_7_-6’SLN (a) Mass spectrum of CD-(Mal-PEG8)_7_-COOH before sugar modification. (b) ^1^H NMR of CD-(Mal-PEG8)_7_-6’SLN in D_2_O. Average number of 6’SLN (3.4) per β-cyclodextrin was calculated by comparing the integral of a distinctive peak from trisaccharide (blue arrow) and the integral of a peak from β-cyclodextrin (red arrow). Both peaks represent 1 hydrogen.
